# Cultivar-dependent and drought-induced modulation of secondary metabolites, adaptative defense in *Fagopyrum esculentum* L

**DOI:** 10.1007/s12298-023-01376-8

**Published:** 2023-11-09

**Authors:** Sytar Oksana, Kovar Marek, Brestic Marian, Zivcak Marek

**Affiliations:** Institute of Plant and Environmental Sciences, Faculty of Agrobiology and Food Resources, Slovak University of Agriculture, Nitra, 94976 Slovakia

**Keywords:** Drought stress, Common buckwheat, Flavonoid, Phenolic acid, Rutin, Chlorogenic acid, Ferulic acid response

## Abstract

The present study investigates the biochemical responses of buckwheat to drought stress, particularly focusing on phenolic acids and flavonoids, abundant in this crop. We hypothesize that distinct genotypic responses to drought stress will lead to variations in phenolic acid accumulation. Two common buckwheat cultivars, Panda (East European origin) and PI 482597 (originating from Zimbabwe), were subjected to drought treatment, with biochemical traits, relative water content, and photosynthetic pigments regularly assessed. While chlorophyll content remained unaffected by dehydration, total carotenoid content decreased. The unique increase in the chlorophyll to carotenoid ratio suggests a specific role of carotenoids in buckwheat's metabolic stress response. While most phenolic acids and flavonoids exhibited increasing trends during progressive dehydration, their dynamics differed. Notably, rutin content increased early in drought stress, while chlorogenic acid and kaempferol showed enhanced levels only under severe dehydration. Genotypic differences were observed in chlorogenic acid, neochlorogenic acid, cryptochlorogenic acid, 4-hydroxybenzoic acid, and quercetin. Conversely, trans-p-coumaric acid, trans-ferulic acid, vanillic acid, rutin, and kaempferol showed similar trends in both cultivars. By aligning observed drought-induced changes in phenolic compound contents with biosynthesis pathways, trade-offs between individual compounds were identified, contributing to the mechanistic understanding of varied stress responses.

## Introduction

Natural antioxidants found in buckwheat plants, including specific phenolic acids, flavonoids, and vitamins, play a crucial role in food production by combating reactive oxygen species (ROS) in the human body (Xu et al. 2017; Sofi et al. 2022; Hajam et al. 2023). These secondary metabolites with antioxidant potential offer protection against various illnesses like atherosclerosis, cancer, emphysema, arthritis, neurodegenerative and cardiovascular diseases, and retinopathy (Liu et al. 2018; Seca and Pinto 2019; Karakaya et al. 2021). Scientific evidence indicates that environmental stresses, particularly drought stress, can elevate the levels of phenolics, flavonoids, vitamins, and antioxidant potential in different plant crops (Bhardwaj et al. 2017; Sarker and Oba 2018; Yang et al. 2020; Albuquerque et al. 2021), with flavonol glycoside synthesis playing a role in drought/osmotic stress resistance through flavonoid biosynthesis regulation (Baozhu et al. 2022).

Addressing the need for stress-resistant genotypes and sustainable yield in water-deficient conditions, the adoption of breeding strategies and cultivation of neglected crops is of paramount importance (Rosero et al. 2020). Despite being a minor crop, buckwheat holds significance in certain countries and is gaining interest in non-traditional regions, contributing to agrobiodiversity and changing consumer preferences (Donno and Turrini 2020). Common buckwheat cultivars, widely grown in Eastern Europe, China, and India (Sytar et al. 2016a, b), contain biologically active compounds that can enhance dietary health.

Compared to Tartary buckwheat (*Fagopyrum tataricum*), common buckwheat (*Fagopyrum esculentum*) generally exhibits lower water deficit resistance but shows drought avoidance traits, while *F. tataricum* displays drought tolerance (Aubert et al. 2021). The interplay between UV-B radiation and drought tolerance in buckwheat has also been documented, highlighting the role of UV-B responsive compounds, particularly phenolics, in buckwheat's response to drought (Germ et al. 2013).

Buckwheat cultivars from different origins, both *F. esculentum* and *F. tataricum,* exhibit variations in phenolic and flavonoid content (Sytar et al. 2014; Mikulajová et al. 2016). Genetic backgrounds influence phenolic acid content in grains, leaves, and flowers of *F. esculentum* (Vollmannová et al. 2021a, b). While limited, evidence suggests that buckwheat cultivars of African origin might respond differently to drought compared to those cultivated in Central and Eastern Europe. Beyond general stress responses, the composition of phenolic compounds in response to stress may differ among accessions of diverse origins. This study aims to investigate the impact of varying levels of dehydration on physiological, biochemical, and photosynthetic responses in buckwheat cultivars of European and African origins, with a specific focus on phenolic acid and flavonoid composition.

## Material and methods

### Plant growth conditions

The buckwheat was cultivated at the PlantScreen™ phenotyping platform (PSI, Drasov, Czech Republic). The cultivation was conducted within a meticulously controlled environment, employing an advanced LED-based lighting system. This lighting configuration encompassed warm white LEDs specially designed for plant cultivation, supplemented by red and far-red LEDs. This combination of LEDs optimized the light spectra to foster ideal plant growth and development. The illumination intensity reached 1100 µmol m^−2^ s^−1^, while the photoperiod adhered to a 16-h day followed by an 8-h night. Temperature conditions alternated between 25 °C during the day and 20 °C at night, with relative humidity levels spanning from 30% during the day to 40% at night.

The watering system functioned automatically, guided by gravimetric measurements to ensure precise regulation. Every two days, the water level within the pots was maintained to achieve 80% of the field water capacity, upholding optimal hydration levels. Introducing drought stress involved a deliberate suspension of soil substrate irrigation, leading to a gradual reduction in water content to 40% of the field water capacity.

For the experimental trials, two distinct cultivars of common buckwheat (*Fagopyrum esculentum*) were selected. The first was the Panda cultivar originating from East Europe, and the second was the PI 482597 cultivar with its origins traced back to Zimbabwe. Vital attributes of these examined genotypes were sourced from the Storage and Documentation of Genetic Resources Genebank at the Crop Research Institute in Prague, Czech Republic.

Grains were planted in pots with 930 g Klasmann TS 3 soil (soil composition: EDTA-chelated Fe, 140 mg L^−1^ N, 160 mg L^−1^ P_2_O_5_, 180 mg L^−1^ K_2_O, 100 mg L^−1^ Mg, micronutrients, pH (H_2_O) 6.0; Klasmann-Deilmann GmbH, Geeste, Germany). Plant germination was initiated within a controlled greenhouse environment (June 2021). After the germination phase, buckwheat sprouts in individual pots were transferred to the PlantScreen™ phenotyping system. Each pot was meticulously labeled with barcodes using the Photon System Instruments software. The pots were then placed on moving belts within the phenotyping system. Over a span of 14 days, the plants underwent cultivation under standard conditions. During this period, an automated phenotyping protocol (not elaborated in this manuscript) was employed for analysis. To facilitate optimal growth of buckwheat seedlings, the pots were regularly watered to maintain 80% of the field water capacity. Approximately 20 days after sowing, all plants had progressed to the fourth leaf stage, characterized by substantial solidity and size, which rendered them suitable for assessments and subsequent drought stress treatment. After reaching the desired leaf stage, different watering treatments were applied to the buckwheat genotypes. These treatments encompassed full watering at 80% field capacity (control) and inducing water stress by gradually reducing watering to as low as 40% of the field water capacity. Leaf samples were systematically collected on distinct days: 20, 25, 28, 32, and 35 days after sowing (DAS), respectively. These correspond to dehydration periods of 1, 5, 8, 12, and 15 days, respectively. To ensure robust results, each genotype was represented by 4–7 replicates, constituting biological replications.

### Extract preparation

The dried plant material, weighing 2 g, was homogenized. Such leaf material was extracted using 20 mL of 80% aqueous methanol (v/v) for a duration of 8 h. The extraction process was carried out on a horizontal shaker at 250 rpm. The extraction process utilized a Nimax 2010 system (Heidolph Instruments, GmbH, Schwabach, Germany). The resulting extract was filtered through Munktell No. 390 filter paper (Munktell & Filtrak GmbH, Bärenstein, Germany). Finally, the extract was stored within 20 mL polyethylene (PE) vials.

### Preparation of calibration solutions and samples

5 mg of each compound was dissolved in 10 mL of methanol (HPLC grade) to prepare single-component standard solutions. The samples were prepared via extraction of 0.05 g of dried milled leaves in 2 mL 80% methanol (V/V). The extraction process was done using horizontal shaker for 4 h. Further step was the filtration of extract to the epindorphes. The sample extracts were stored in the sampler manager at 4 °C. Before the planned HPLC analysis; the experimental extract was purified via syringe PTFE filters (0.45 µm, 25 mm) (Agilent Technologies, Waldbronn, Germany).

### HPLC–DAD determination of selected phenolic compounds

The HPLC method utilized in this study was initially developed by Germ et al. (2019). The detection of specific phenolic compounds in the experimental extracts was conducted using an HPLC–DAD Agilent 1260 system (Agilent Technologies, Waldbronn, Germany). A C18 reverse-phase Cortecs column (4 mm × 250 mm × 5 µm) from Merck KGaA (Darmstadt, Germany) was employed for all experimental analyses.

For HPLC analysis, analytical standards, chemicals, and solvents were procured from Sigma-Aldrich (St. Louis, MO, USA). The mobile phase composition consisted of acetonitrile (phase A) and deionized H_2_O with 0.1% phosphoric acid (phase B), adjusted to a pH of 3.5 with phosphoric acid. Sample injection volume was set at 5 µL. Spectral scanning ranged from 210 to 400 nm. Phenolic acids were identified at a wavelength of 320 nm, while flavonoids were detected at 372 nm. Spectral data analysis was facilitated by employing Agilent OpenLab ChemStation software for LC 3D Systems.

### Determination of the relative water content in leaves

The leaf relative water content (RWC) study was performed to estimate the levels of dehydration. To exclude the damage to plant tissues, measurements were limited to stage when the visual symptoms of drought stress arose, the RWC was estimated. The fresh weight (FW) of flag leaves samples were analyzed. The leaf parts were put in distilled water for a period of 4 h and the turgor weight (TW) of the leaf was estimated. To get the dry weight (DW) measurements, the leaves were kept in an oven at 80 °C for 3 h. The calculation of RWC was done using the following formula:

$${\text{RWC }}\left( \% \right) \, = \, \frac{{\left[ {{\text{FW }}{-}{\text{DW}}} \right] \, \times \, 100}}{{\left[ {{\text{TW }}{-}{\text{DW}}} \right]}}$$

### Determination of photosynthetic pigments in leaves

Five leaf discs with a diameter of 7 mm were meticulously excised from fully developed and expanded buckwheat leaves. These segments were then subjected to homogenization through a combination of sea sand, MgCO_3_, and 100% acetone. The ensuing step involved the extraction of photosynthetic pigments utilizing an 80% acetone (v/v) solution.

After extraction, the obtained mixture was subjected to centrifugation at 1500 rpm for 2 min. Subsequently, the solution's absorbance was measured at 470 nm, 647 nm, and 663 nm. To account for scattering, readings were also taken at 750 nm. This measurement was carried out using a UV–VIS spectrophotometer model Jenway 6405 (Jenway, UK).

The concentrations of specific pigments including chlorophyll a (Chl a), chlorophyll b (Chl b), and total carotenoids (CarX) were determined by applying Lichtenthaler's Eqs. (1987). The quantified values were then expressed in units of mg m^−2^ leaf area.

### Statistical analysis

Plant arrangement in the cultivation area of PlantScreenTM phenotyping platform was full randomized. All data were presented as the mean values, with ± indicating the standard error of the mean (SEM) and the range of minimum to maximum values also provided. Statistical analysis was performed using the Statistica program (version 10 for Windows; Tulsa, OK, USA). Normal distribution and homogeneity of experimental dataset were tested using Kolmogorov–Smirnov and Lavene´s statistical test, respectively. Linear regressions between RWC and contents of chemical compounds were performed using method of least squares and coefficient of determination (R^2^) and standard error (S.E.) were calculated.

To evaluate the statistical differences between cultivars, a one-way ANOVA test was conducted, followed by the Tukey post hoc test for multiple comparisons. The threshold for statistical significance was set at *P* ≤ 0.05 (*), *P* ≤ 0.01 (**), *P* ≤ 0.001 (***), and *P* ≤ 0.0001 (****).

## Results

**Fig. 1:**
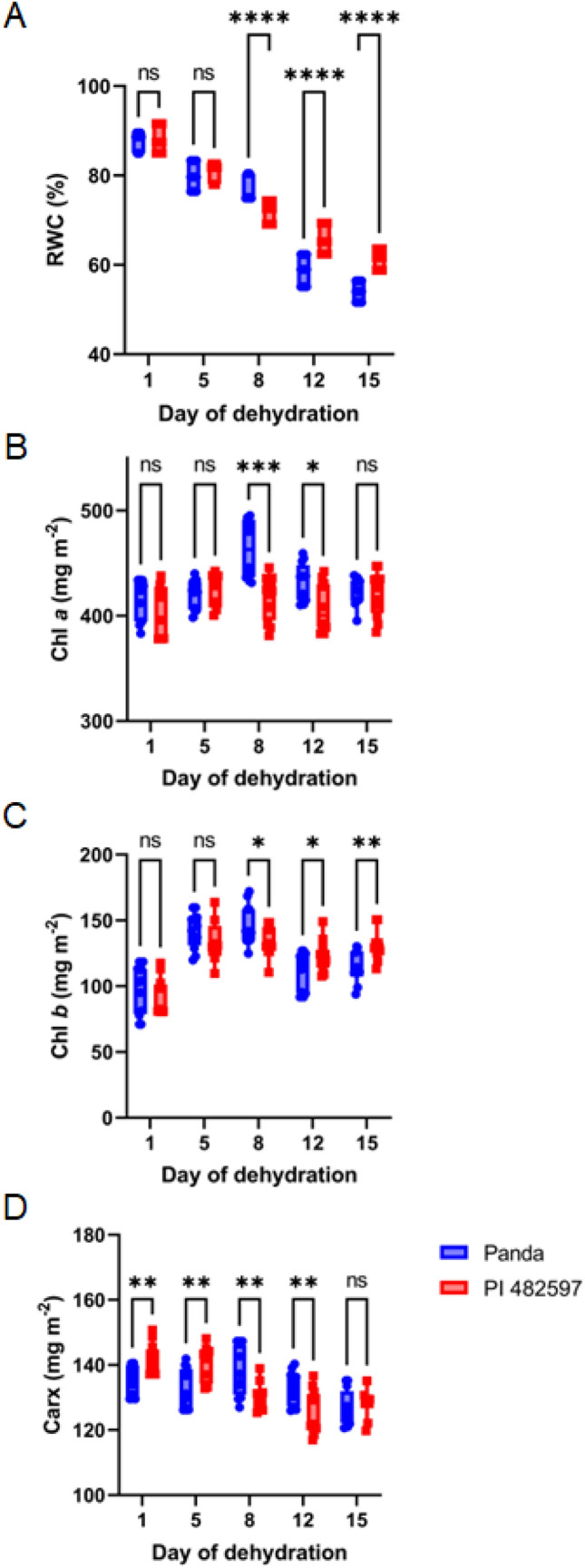
RWC value (**A**), chlorophyll *a* (**B**), chlorophyll *b* (**C**) and total carotenoids (**D**) contents under drought stress in the leaves of experimental buckwheat cultivars—Panda and PI 482597. Each box and whiskers represent the mean and min–max values of all 12 individual samples. The level of statistical significance of differences between average values measured in individual genotypes was tested by unpaired t-test and indicated as non-significant (ns) or significant at *p* < 0.05 (*),*p* < 0.01 (**), *p* < 0.001 (***) and *p* < 0.0001 (****)

To describe the effect of drought stress, measurements of leaf RWC during all days of leaf samples collection of the experiment were performed. The RWC of the leaves of both experimental buckwheat cultivars tended to decrease under continuous drought stress. The decline in dehydration level was more significant with the duration of the experiment from 25 till 36 DAS. The highest dehydration level was observed on the 15th day of dehydration at the end of the investigation. The buckwheat cultivar Panda was characterized by a more than 60% dehydration level compared to the cultivar PI 482597, indicating a faster water loss (Fig. 1).

In the current study, both analyzed buckwheat cultivars maintained chlorophyll *a* concentration at control levels and exhibited a slight increase in chlorophyll *b* concentration, ranging between 25 and 30% (Fig. 1). On the contrary, we observed a slight decrease of carotenoids in PI 482597, while the values in cultivar Panda remained stable. The decrease of carotenoids level in the leaves of buckwheat was also recorded in a previous study (Mibei et al. 2016).

**Fig. 2:**
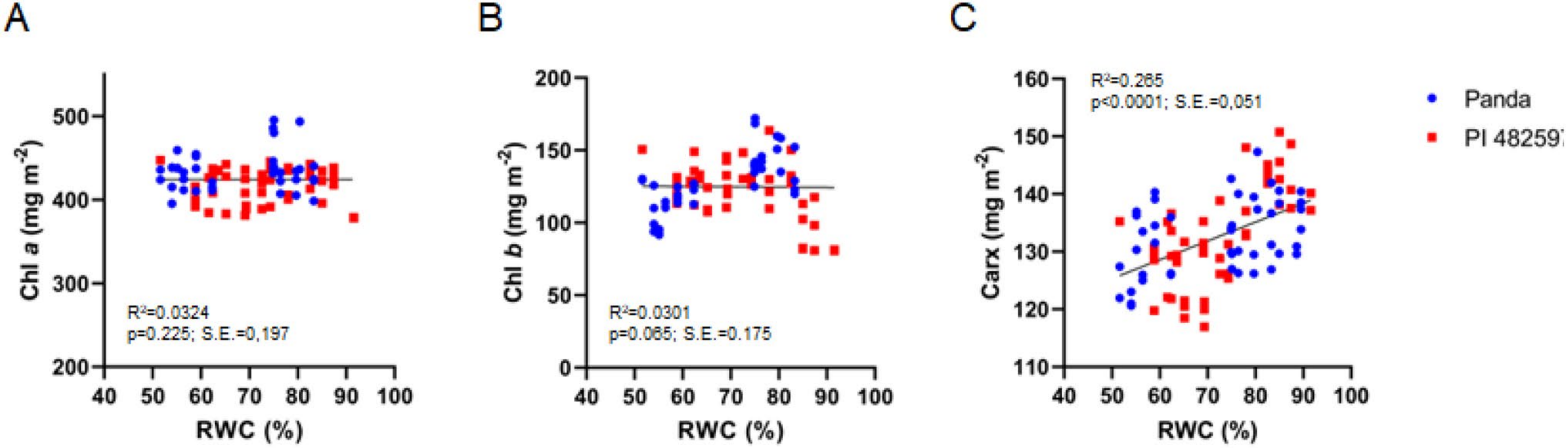
Relationship of chlorophyll *a* (**A**), chlorophyll *b* (**B**) and total carotenoids (**C**) content to RWC during drought stress in leaves of both cultivars Panda and PI 482597. The solid line shows linear regression of the relationship using least squares method. R^2^—coefficient of determination, *p*—probability, S.E.—standard error

The trends of the relationship between chlorophylls vs. RWC and carotenoids vs. RWC indicate differences in acclimation strategies at the level of photosynthetic structures between the two genotypes (Fig. 2). Increasing trend of carotenoids vs. RWC under drought treatment was observed in both buckwheat cultivars. The ratio chlorophyll vs. RWC mainly remained unchanged in drought-stressed buckwheat cultivars.

**Fig. 3:**
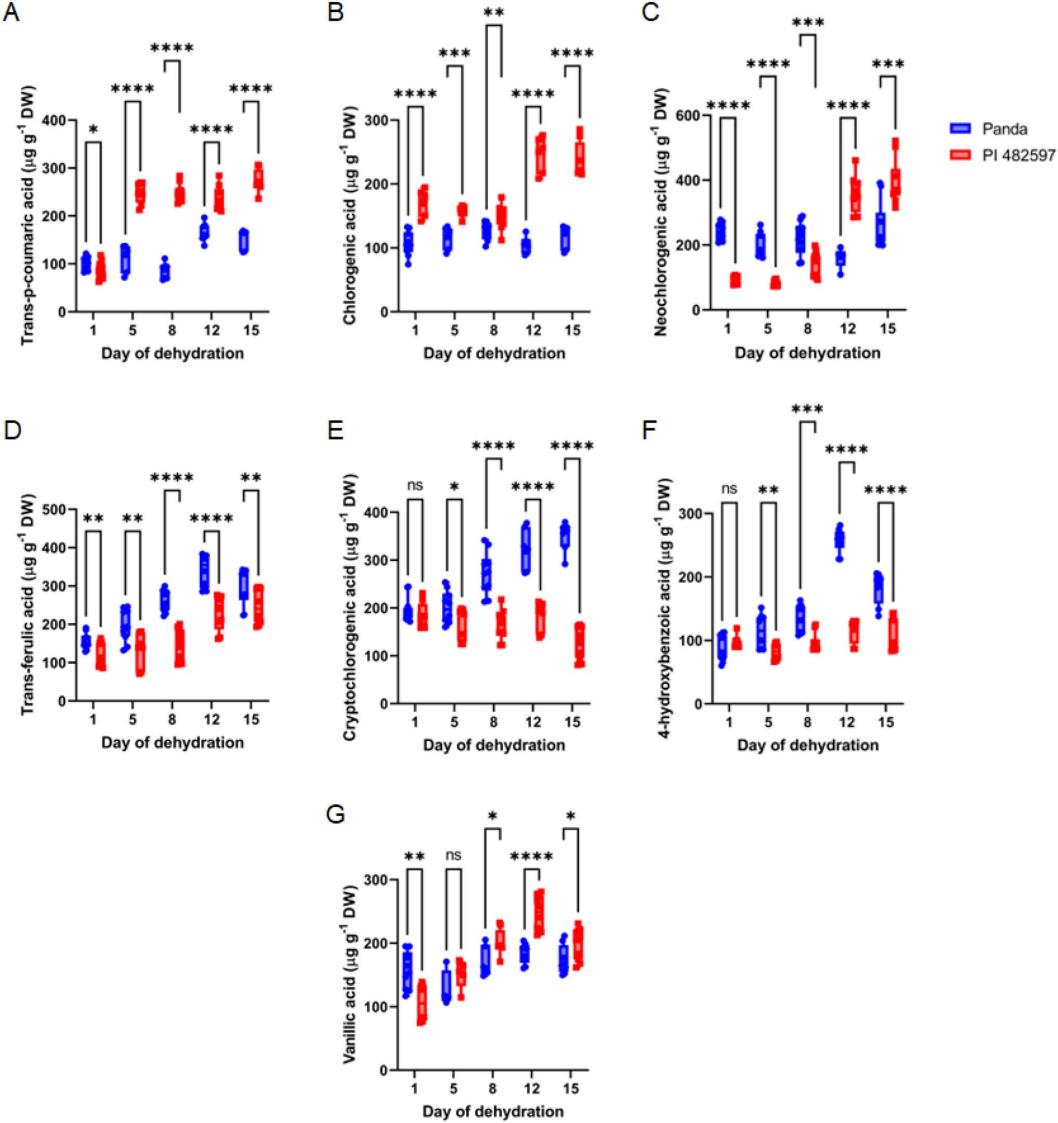
The content of seven identified phenolic acids (trans-p-coumaric (A), chlorogenic (**B**), neochlorogenic (**C**), trans-ferulic (**D**), cryptochlorogenic (**E**), 4-hydroxybenzoic (**F**) and vanillic (**G**) acids) in the buckwheat cultivars of different origin (Panda and PI 482597) under progressive drought stress. Each box and whiskers represent the mean and min–max values of all 12 individual samples. The level of statistical significance of differences between average values measured in individual genotypes was tested by unpaired t-test and indicated as non-significant (ns) or significant at *p* < 0.05 (*),*p* < 0.01 (**), *p* < 0.001 (***) and *p* < 0.0001 (****)

Considering the contents of phenolic compounds, three types of chlorogenic acids were identified in the experimental buckwheat cultivars—cryptochlorogenic acid (4-O-caffeoylquinic acid), chlorogenic acid (5-O-caffeoylquinic acid), neochlorogenic acid (3-O-caffeoylquinic acid). Under drought stress, we observed some changes in the level of chlorogenic acid formation (Fig. 3). The alterations in the concentrations of neochlorogenic acid (3-O-caffeoylquinic acid) and chlorogenic acid (4-O-caffeoylquinic acid) were largely consistent between the two buckwheat cultivars under investigation. Notably, their content exhibited a substantial increase in the cultivar PI 482597, particularly toward the culmination of the experimental timeline when the plants experienced intense dehydration (on the 12th and 15th days of dehydration).

Contrarily, within the buckwheat cultivar Panda, the levels of neochlorogenic acid (3-O-caffeoylquinic acid) and chlorogenic acid (4-O-caffeoylquinic acid) remained relatively stable at control levels. Interestingly, the content of cryptochlorogenic acid (4-O-caffeoylquinic acid) witnessed a significant rise in the continuously drought-stressed Panda, though this was not mirrored in the PI 482597 cultivar.

Furthermore, the East European cultivar Panda demonstrated elevated concentrations of neochlorogenic acid (3-O-caffeoylquinic acid) and chlorogenic acid, particularly during the early stages of drought stress, compared to the African cultivar PI 482597.

The correlation of the chlorogenic acids forms (chlorogenic acid, neochlorogenic acid, cryptochlorogenic acid) vs. RWC in the buckwheat cultivars of different origins under drought stress showed a direct correlation between the level of identified chlorogenic acid forms and RWC (Fig. 6). The differences in the trends of chlorogenic acid forms under drought stress in the two buckwheat cultivars were evident.

**Fig. 4:**
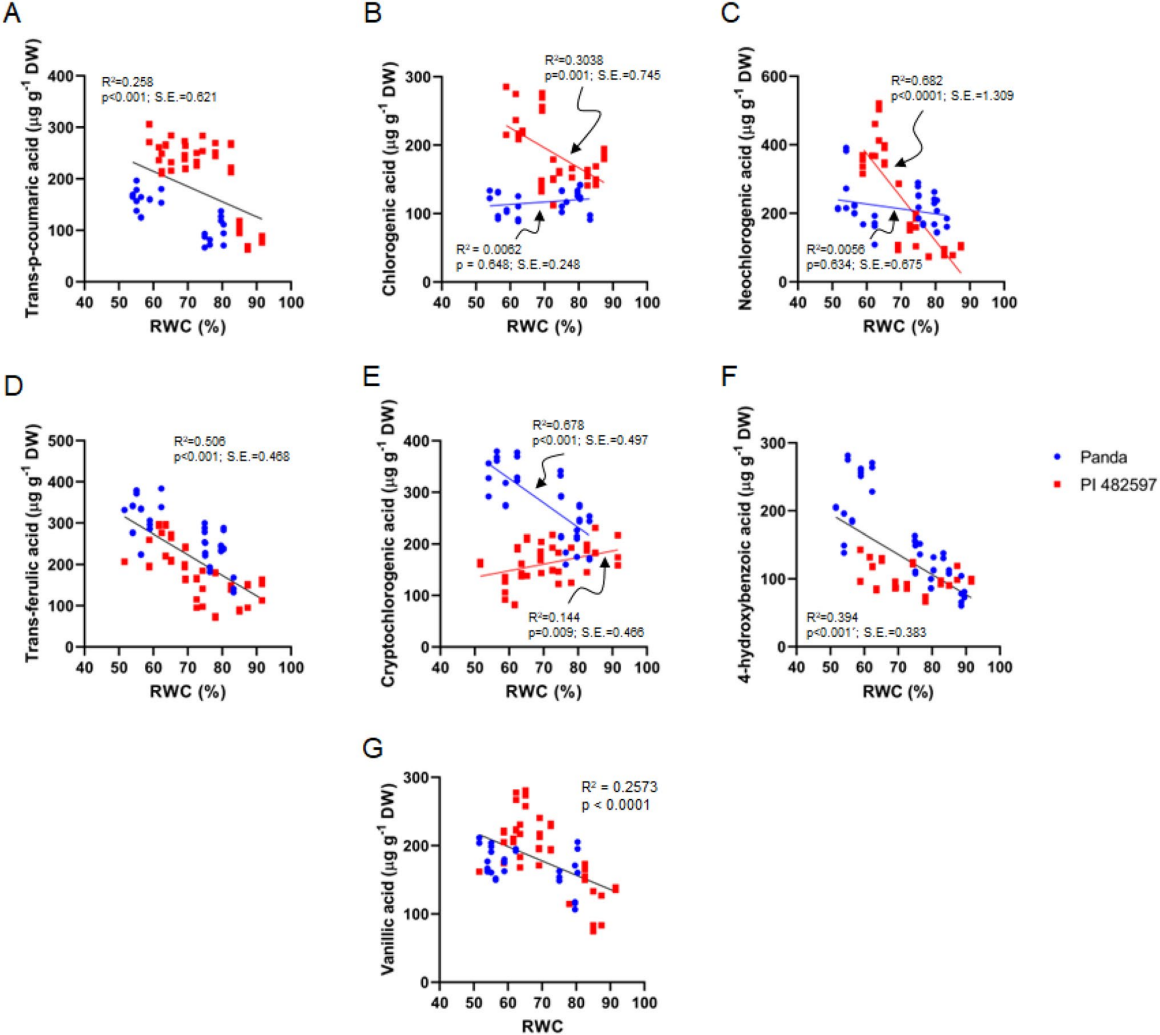
Relationship between the trans-p-coumaric (**A**), chlorogenic (**B**),neochlorogenic (**C**), trans-ferulic (**D**), cryptochlorogenic (E), 4-hydroxybenzoic (F), and vanillic (G) acids content and the leaf RWC during drought stress in leaves of both cultivars Panda and PI 482597. The solid line shows linear regression of the relationship using least squares method. R^2^—coefficient of determination, *p*—probability, S.E.—standard error

A noteworthy twofold increase in the content of 4-hydroxybenzoic acid was discernible in the Panda cultivar specifically on the 12th day of dehydration, when juxtaposed with the instances of 8 or 15 days of drought-induced dehydration (as depicted in Fig. 4). Intriguingly, in the case of the buckwheat cultivar PI 482597, an early response to drought stress was not accompanied by alterations in the level of 4-hydroxybenzoic acid.

Furthermore, the vanillic acid content in the leaves of cultivar PI 482597 exhibited a propensity to elevate until the 12th day of dehydration. In contrast, within the leaves of the Panda cultivar, the vanillic acid content remained consistent at control levels throughout the entire experimental duration.

Amidst the context of drought stress, the alterations in hydroxybenzoic acids, specifically 4-hydroxybenzoic and vanillic acids, exhibited divergence between the two buckwheat cultivars. Notably, the correlation analysis between hydroxybenzoic acids (4-hydroxybenzoic acid and vanillic acid) and Relative Water Content (RWC %) revealed a robust association with the extent of dehydration (Fig. 4). A significantly higher level of trans-*p*-coumaric acid during all stages of drought treatment in the buckwheat cultivar of African origin was observed. At the same time, the tendency to raise the trans-p-coumaric acid content in the leaves of East European buckwheat cultivar was observed only 12th to 15th days after the start of dehydration (Fig. 4A).

Subsequently, commencing from the 8th day of dehydration, there was a noticeable augmentation in the concentration of trans-ferulic acid within the Panda cultivar. In contrast, the PI 482597 cultivar demonstrated an elevation in ferulic acid content specifically on the 12th and 15th days of the dehydration period (as illustrated in Fig. 4D).

**Fig. 5:**
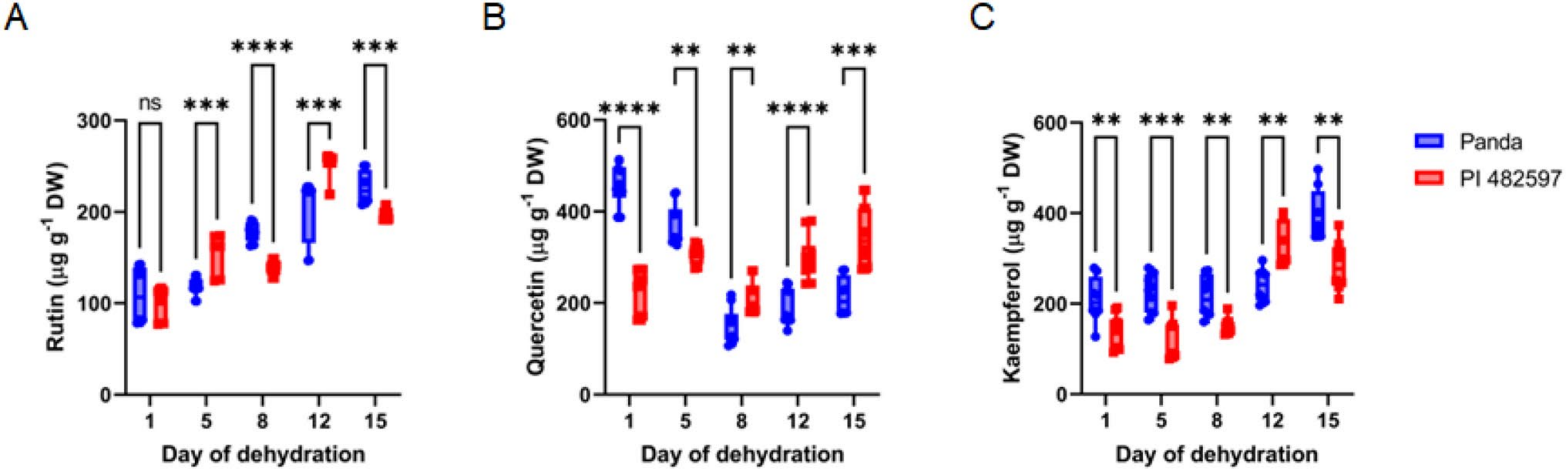
The content of flavonoids rutin (**A**), quercetin (**B**), and kaempferol (**C**) in the buckwheat cultivars of different origins (Panda and PI 482597) under drought stress. Each box and whiskers represent the mean and min–max values of all 12 samples. The level of statistical significance of differences between average values measured in individual genotypes was tested by unpaired t-test and indicated as non-significant (ns) or significant at *p* < 0.05 (*),*p* < 0.01 (**), *p* < 0.001 (***) and *p* < 0.0001 (****)

**Fig. 6:**
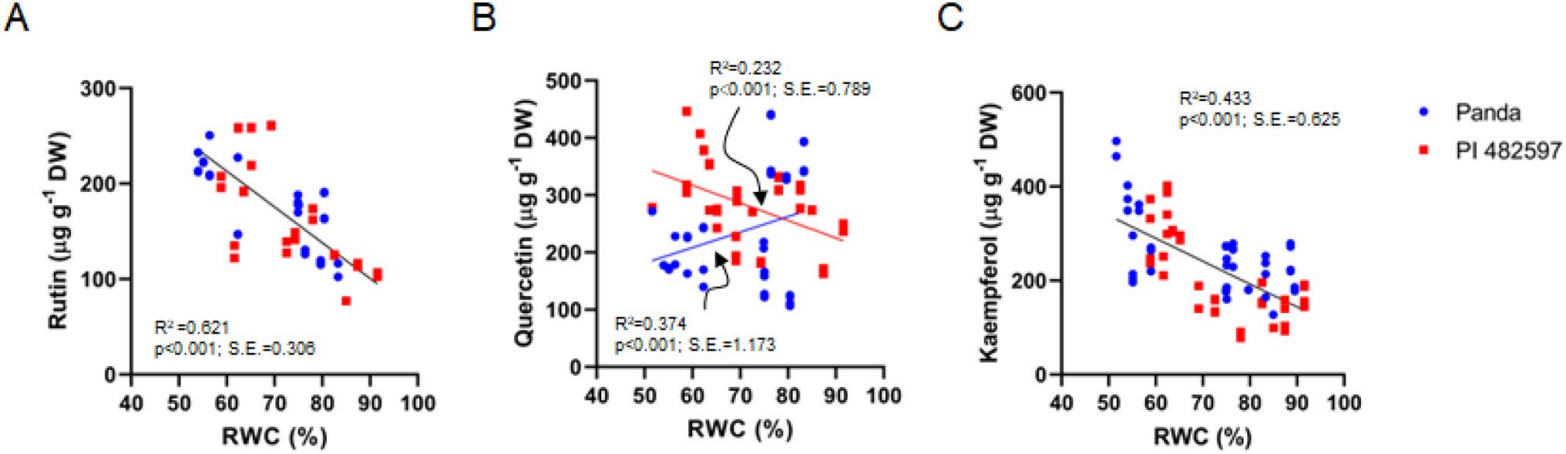
Relationship of rutin (**A**), quercetin (**B**), Kaempferol (**C**) contents and RWC during drought stress in leaves of both cultivars Panda and PI 482597. The solid line shows linear regression of the relationship using least squares method. R^2^—coefficient of determination, *p*—probability, S.E.—standard error

The data of the presented experiment showed a significantly increased level of rutin in both experimental buckwheat cultivars compared to the first day of dehydration (Fig. 5A). In the case of quercetin (Fig. 5B), we observed the contrasting response in two explored genotypes: the decreasing level in leaves of Panda cultivar and the significantly enhanced level in PI 482597. The contents of kaempferol (Fig. 5C) increased significantly only in the last two measurements (days 12–15). The relationships between the rutin and kaempferol contents and RWC (Fig. 6 A and C) were mostly linear, indicating a similar increase with decreasing water content in both genotypes.The response of quercetin content to dehydration differed between the two cultivars. In cultivar Panda, quercetin content increased with dehydration, while in cultivar PI 482597, there was a sharp decrease in quercetin content when relative water content (RWC) fell below 80%, showing a non-gradual decline at lower RWC levels.

## Discussion

Drought stress, accompanied by a gradual reduction in leaf water content, typically triggers physiological responses at various levels. These responses encompass biochemical changes, which could arise from drought-induced metabolic disruptions or serve as an expression of protective mechanisms within plant cells. The focus of our study was to evaluate the levels of photosynthetic pigments, particularly chlorophylls, to gauge the impact of progressive dehydration on the photosynthetic apparatus. While some plants exhibit substantial declines in chlorophyll and carotenoid levels under drought stress (Mibei et al. 2016; Raja et al. 2020), variations in chlorophyll and carotenoid responses are influenced by plant sensitivity to drought stress (Rustioni and Bianchi 2021). Interestingly, some instances, such as water stress in tataricum buckwheat, can lead to augmented chlorophyll content (Aubert and Quinet 2022).

In our investigation, chlorophyll *a* remained relatively stable amid dehydration, whereas a marginal decrease was observed in chlorophyll b levels within the Panda genotype. Conversely, the African-origin PI 482597 genotype exhibited heightened chlorophyll b content, indicating potentially elevated stress acclimation and reduced photosynthetic apparatus damage. These findings deviate from outcomes reported for buckwheat tataricum (Aubert et al. 2021).

Distinctly, we observed a significant reduction in carotenoid levels during progressive drought stress. This finding is intriguing as chlorophylls generally exhibit higher sensitivity to drought than carotenoids, and the chlorophyll-to-carotenoid ratio typically decreases under stress conditions (Zhang et al. 2008). In our experiment, a stable chlorophyll content accompanied by decreasing carotenoids led to an apparent increase in the chlorophyll-to-carotenoid ratio. Carotenoid content is governed by the equilibrium between biosynthesis and degradation (Nisar et al. 2015). Given that the decline in carotenoids didn't correspond to reduced chlorophylls, it seems to be part of an acclimation response rather than mere damage. This carotenoid cleavage might trigger the production of hormones, signals, and volatile compounds, all significant components in drought stress response (Nisar et al. 2015).

Although our study doesn't unravel the metabolic intricacies of carotenoid transformation, it highlights buckwheat as a promising model for exploring a unique carotenoid-related mechanism of plant stress defense. This aspect warrants deeper exploration in future research endeavors.

Our results encompassed the presence of various phenolic compounds in the leaves of both buckwheat cultivars. Among the flavonoids, quercetin content was notably highest. The phenolic acid composition unveiled elevated levels of trans-*p*-coumaric acid in cv. Panda, while cv. PI 482597 exhibited predominance of trans-ferulic acid. Neochlorogenic acid stood out as the prominent chlorogenic acid form in both cultivars. The dynamics of phenolic content exhibited diversity under drought stress between the two genotypes, signifying distinct adaptive stress responses.

Earlier investigations on buckwheat emphasized the impact of abiotic stress on phenolic compounds, particularly the increased levels of quercetin and rutin under salinity (Kim et al. 2019). Similar patterns were evident in other phenolic-rich plants, such as enhanced chlorogenic acid levels in Achillea pachycephala under drought stress (Gharibi et al. 2019; Al-Ghamdi and Elansary 2018), and Hypericum pruinatum, a salt-tolerant species (Caliskan et al. 2017). Phenolics are believed to have a crucial defense role in drought tolerance (Nouraei et al. 2018; Moreira et al. 2020). Research indicates that phenolic acids, including hydroxybenzoic acids and hydroxycinnamic acids, respond to water scarcity (Sarker and Oba 2018). Our study further demonstrates diverse effects of dehydration levels on individual phenolic compounds.

Phenolic acids can act as antioxidants, scavenging reactive oxygen species (ROS) during abiotic stress (Bistgani et al. 2019; Chen et al. 2019). Activation of the phenylpropanoid biosynthetic pathway during abiotic stress leads to increased phenolic acid synthesis, including cinamylmalic, caffeic, gallic, ferulic, and vanillic acids (Bistgani et al. 2019; Al-Ghamdi and Elansary 2018). Key genes in this pathway, such as chalcone synthase (CHS) and phenylalanine ammonia-lyase (PAL), become upregulated under abiotic stress, resulting in enhanced phenolic biosynthesis (Sharma et al. 2016, 2019) and improved abiotic stress tolerance (Wang et al. 2019).

Intriguingly, hydroxycinnamic acids (trans-ferulic, chlorogenic, trans-p-coumaric acids) notably increased in leaves of both cultivars, albeit cv. PI 482597 exhibited higher levels of chlorogenic acid and trans-ferulic acid at the early drought stress stages compared to cv. Panda. These trends were consistent with the outcomes presented in Figs. 4 and 9.

Previously, the influence of cultivars on trans-caffeic, chlorogenic, and trans-sinapic acids, as well as flavonoids vitexin and kaempferol, in buckwheat crops was explored. Varied phenolic acid quantities in different parts of common buckwheat cultivars from distinct origins have indicated genetic factors regulating phenolic compound biosynthesis (Vollmannová et al. 2021a, b). Enhanced chlorogenic acid accumulation, along with related esters involved in lignin biosynthesis, is recognized under abiotic stress in several plant species (Wang et al. 2019; Sarker and Oba 2018). Chlorogenic acids, arising from esterification of caffeic, ferulic, and p-coumaric acids with quinic acid can exist in diverse isomeric forms due to structural variations (Soviguidi et al. 2022). Vicinal hydroxyl groups on aromatic residues characterize chlorogenic acids (Liang and Kitts 2015). Notably, chlorogenic acid exhibited augmentation since the 12th day of dehydration, suggesting a gradual increase.

Neochlorogenic and chlorogenic acid fractions exhibit potential scavenging of reactive oxygen species (ROS) (Thurow 2012). High levels of caffeic acid in certain medicinal plants correspond to increased antioxidant potential (Tajner-Czopek et al. 2020). The content of cryptochlorogenic acid (4-O-caffeoylquinic acid) increased significantly in the Panda cultivar during continuous drought stress, whereas the Zimbabwe-origin cultivar maintained control-level content throughout dehydration. The East European cultivar, Panda, demonstrated elevated levels of neochlorogenic and chlorogenic acids during the earlier stages of drought treatment compared to PI 482597, signifying their potential role in adaptive stress responses.

Antioxidant potential of phenolic acids and their derivatives often correlates with hydroxyl group numbers (Samec et al. 2021). The carboxyl group alone lacks electron-scavenging abilities, but deprotonated carboxyl enhances electron donation, favoring H atom relocations, leading to electron-donating radical scavenging activity (Hendrickson et al. 1994). A higher count of hydroxyl groups and methoxylation enhances antioxidant potential (e.g., intensified ferulic acid synthesis proves more defensive than p-coumaric acid) (Rice-Evans et al. 1996). Ferulic acid exhibits versatility and responsive scavenging of free radicals, inhibiting enzymes related to free radical synthesis (Zduńska et al. 2018). The East European cultivar displayed increased ferulic acid content on the 8th, 12th, and 15th days of dehydration compared to day one, while PI 482597 increased content only in the later stages.

Ferulic acid's covalent bond to cell wall carbohydrates acts as a light filter, mitigating mesophyll penetration during drought stress and modulating leaf growth (Hurá et al. 2009). Trans-ferulic acid increase induces stomatal closure via vascular-to-guard cell signaling, restraining water loss.

In buckwheat, enzymes with low Km values for rutin metabolism indicate specificity for rutin and rutinosidase (Lukšic et al. 2016). Rutin and rutinosidase enhance defense systems against environmental stressors (Suzuki et al. 2015). Both cultivars exhibited shifts in flavonoid rutin levels from the 8th day of dehydration. Rutin content rose in the early drought stress stages, while quercetin content changed on the 8th day, decreasing in the East European cultivar and increasing in PI 482597. Similarly, kaempferol levels changed on later dehydration days (12–15). Biosynthesis of flavonol glycosides like rutin involves flavonols, particularly quercetin (Matsui and Walker 2020), explaining the fluctuations.

Quercetin and kaempferol, both flavonols, aid in mitigating drought stress (Nakabayashi et al. 2014). The significant kaempferol increase in PI 482597 suggests its role in stress acclimation.

Considering biosynthetic pathways, a schematic profile of phenolic acids and flavonoids in examined buckwheat cultivars under drought stress was established (Fig. 8). This outlines *p*-coumaric acid's potential early stress response role. It appears to be a key metabolite in phenolic acid and flavonoid biosynthesis, known for its phytochemical potential in various edible plants. *P*-coumaric acid (4-hydroxy-cinnamic acid) is broadly found in the cell walls of graminaceous plants and is characterized by its ability to decline low-density lipoprotein peroxidation (Boz 2015). *P*-coumaric acid has antioxidative and osmotic stress response capabilities, indicating its significance in cell-wide transcriptional changes (Wang et al. 2022).

**Fig. 7:**
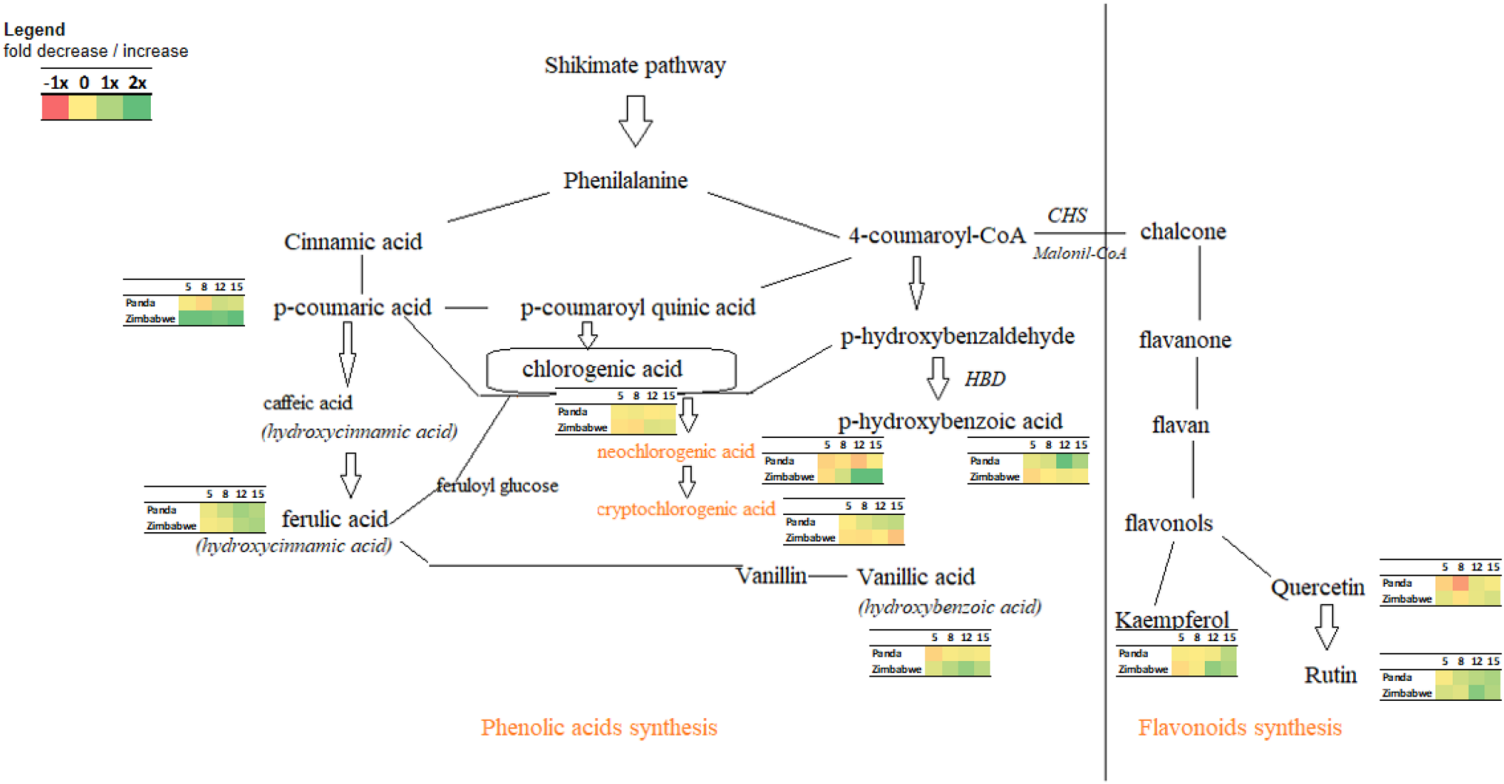
Scheme of biosynthesis of identified phenolic acids and flavonoids with visualization of their values of fold changes in observed buckwheat cultivars Panda (East European origin) and PI 482597 (Zimbabwe) during the period of dehydration

The scheme (Fig. 7) reveals discernible trade-offs between certain members of the analyzed phenolic group across the genotypes. These trade-offs manifest in both individual branch comparisons (e.g., 4-hydroxybenzoic acid vs. vanillic acid) and intra-branch comparisons of metabolites (e.g., neochlorogenic acid vs. cryptochlorogenic acid).

Contrarily, there appears to be no apparent trade-off between the biosynthesis branch of rutin or quercetin and other constituents within the phenolic family.

**Fig. 8:**
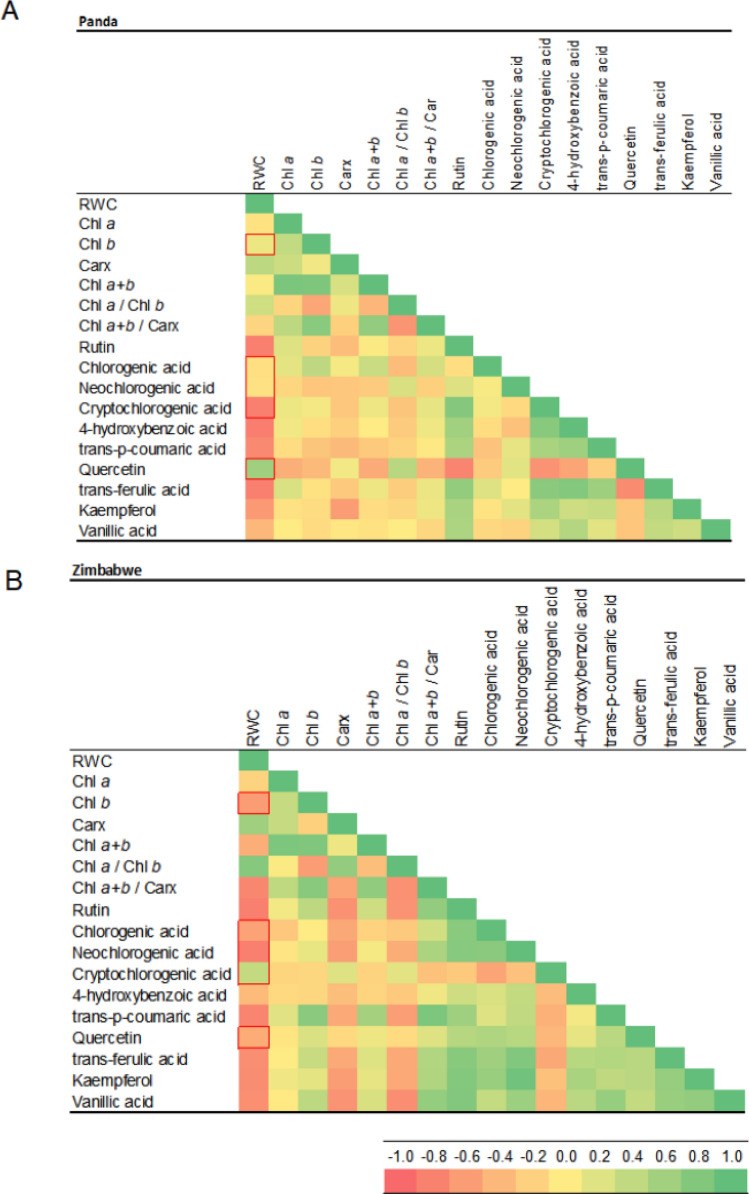
The visualized correlation matrix of analyzed phenolic acids and flavonoids with the characteristic of changes in their content in buckwheat cultivars Panda (East European origin) (**A**) and PI 482597 (Zimbabwe origin) (**B**) during the period of dehydration

Correlation analysis (Fig. 8) revealed positive correlations between rutin and compounds like trans-p-coumaric acid, trans-ferulic acid, vanillic acid, and kaempferol in both buckwheat cultivars. Rutin biosynthesis is interlinked with subsequent steps, involving p-coumaroyl-oA > dehyhydrokaempferol > dihydroquercetin > quercetin > isoquercetin > rutin (Matsui and Walker 2020; Kianersi et al. 2020). Drought stress elicits varied changes in rutin content among different plants—reduction, moderate increase, or significant rise (Lucci and Mazzafera 2009; Yang et al. 2020). Flavonoid accumulation shifts under drought stress were documented in Arabidopsis thaliana (Nakabayashi et al. 2014). Our study displays differing quercetin and rutin alterations in experimental buckwheat cultivars. In Panda, quercetin and rutin levels significantly increased, largely maintaining control levels. In the Zimbabwe-origin cultivars, quercetin decreased, while specific flavonoid levels increased significantly.

The alternation of chlorogenic acids forms in the experimental buckwheat cultivars under drought stress was also observed (Fig. 7). Chlorogenic acids showed an increase in water stress in *Chrysanthemum morifolium* L. as well (Hodaei et al. 2018). Chlorogenic acid had a dynamic trend in response to the drought stress in *Morus alba* L. Variety Yu-711 (Ackah et al. 2021).

Drought may disrupt the shikimate-mediated secondary metabolism system, triggering changes in other metabolic networks (Guo et al. 2018). Salinity stress elevated chlorogenic acid accumulation in honeysuckle (Lonicera japonica Thunb.) (Yan et al. 2016); similarly, drought stress boosted its presence in Achillea pachycephala Rech.f (Gharibi et al. 2019). Limited studies have confirmed drought's enhancement of bioactive compounds, phenolic acids, and flavonoids, yet their accumulation mechanisms remain unclear (Sarker and Oba 2018; Yang et al. 2020; Albuquerque et al. 2021).

**Fig. 9:**
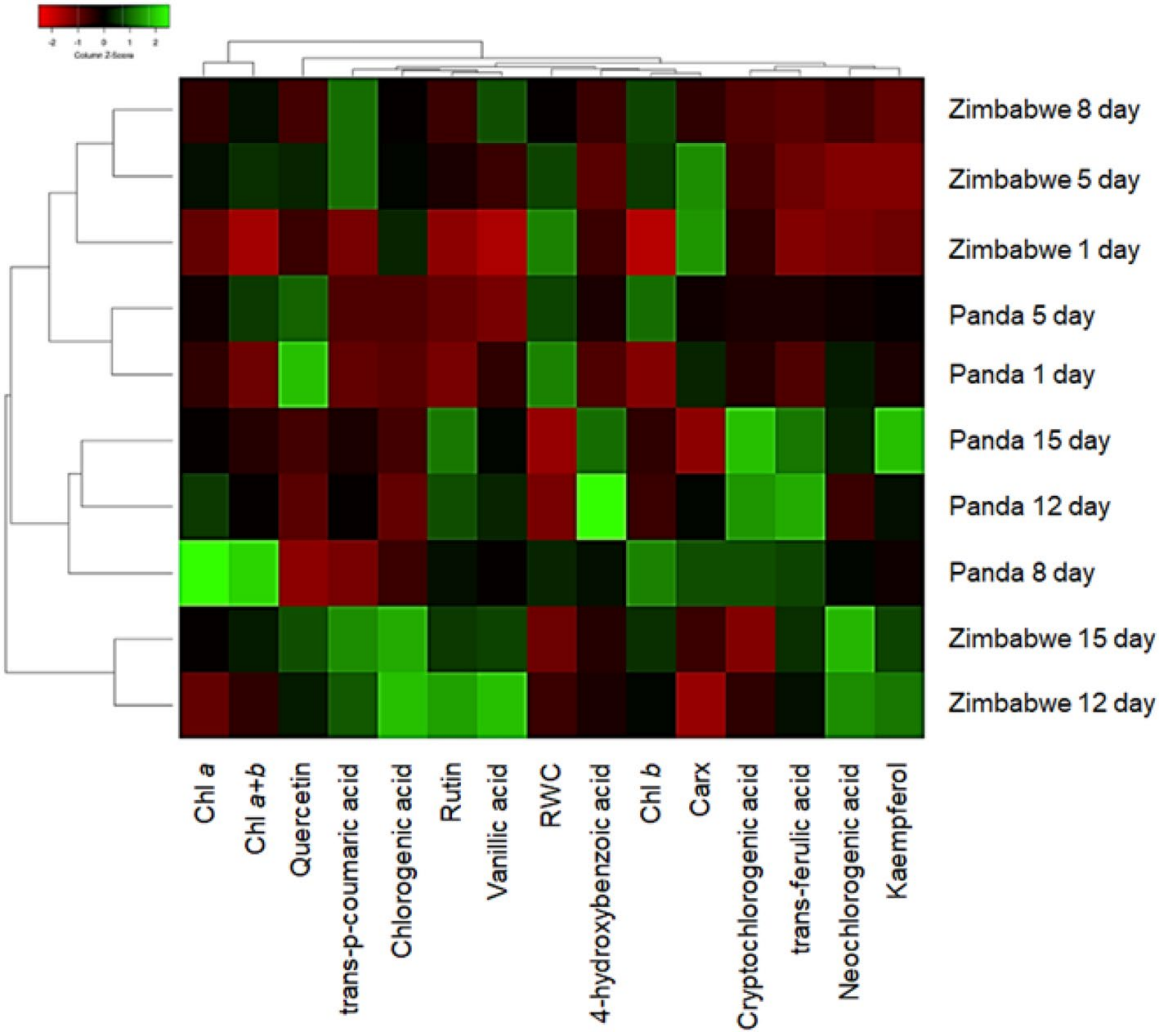
Heatmap of identified pigments, phenolic acids, and flavonoids with the characteristic of changes in their content in buckwheat cultivars Panda (East Europe) and PI 482597 (Zimbabwe) during the period of dehydration

Summarizing effects, we offer a heatmap of relative parameter values on different days (Fig. 9). The dendrogram groups' values based on genotypes, with measurement day being the dominant factor for all compounds. Drought's impact on phenolic compound contents cannot be generalized from a few selected molecules, highlighting the complexity of metabolic responses to environmental constraints and the significance of genotype x environment interaction studies.

## Conclusion

In current study, we compared photosynthetic pigment, phenolic acid, and flavonoid content changes in two common buckwheat genotypes under progressive drought stress. Chlorophyll levels remained stable, while total carotenoid content decreased with dehydration. The elevated chlorophyll to carotenoid ratio was attributed to metabolic carotenoid conversion during stress adaptation. Phenolic acid and flavonoid contents generally increased, with variations in dynamics. Genotypic differences were observed in several compounds. African drought-tolerant cultivar exhibited specific increases in quercetin, neochlorogenic, and chlorogenic acids, unlike the drought-susceptible genotype. These findings shed light on phenolic responses to drought, offering insights into potential trade-offs and genotypic patterns. This study enhances understanding of drought effects on stress-related compounds in buckwheat, suggesting avenues for further comprehensive research across diverse germplasm.

## References

[CR1] Ackah M, Shi Y, Wu M, Wang L, Guo P, Guo L, Jin X, Li S, Zhang Q, Qiu C, Lin Q, Zhao W (2021). Metabolomics response to drought stress in *Morus*
*alba* L variety Yu-711. Plants (basel).

[CR2] Albuquerque BR, Heleno SA, Oliveira MBPP, Barros L, Ferreira ICFR (2021). Phenolic compounds: current industrial applications, limitations and future challenges. Food Funct.

[CR3] Al-Ghamdi AA, Elansary HO (2018). Synergetic effects of 5-aminolevulinic acid and *Ascophyllum*
*nodosum* seaweed extracts on *Asparagus* phenolics and stress related genes under saline irrigation. Plant Physiol Biochem.

[CR4] Aubert L, Quinet M (2022). Comparison of heat and drought stress responses among twelve tartary buckwheat (*Fagopyrum*
*tataricum*) varieties. Plants (basel).

[CR5] Aubert L, Konrádová D, Barris S, Quinet M (2021). Different drought resistance mechanisms between two buckwheat species *Fagopyrum*
*esculentum* and *Fagopyrum*
*tataricum*. Physiol Plant.

[CR6] Baozhu L, Ruonan F, Yanting F, Runan L, Hui Zh, Tingting Ch, Jiong L, Han L, Xiang Zh, Chun-peng S (2022). The flavonoid biosynthesis regulator PFG3 confers drought stress tolerance in plants by promoting flavonoid accumulation. Environ Exp Bot.

[CR8] Bhardwaj RD, Kaur L, Srivastava P (2017) Comparative evaluation of different phenolic acids as priming agents for mitigating drought stress in wheat seedlings. In: Proceeding of National Academy of Sciences, India Section B: Biological Scienes. 87, 1133–1142 10.1007/s40011-015-0690-y

[CR9] Bistgani ZE, Hashemi M, Dacosta M, Craker L, Maggi F, Morshedloo MR (2019). Effect of salinity stress on the physiological characteristics, phenolic compounds and antioxidant activity of *Thymus*
*vulgaris* L. and *Thymus*
*daenensis* Celak. Ind Crop Prod.

[CR10] Boz H (2015). p-Coumaric acid in cereals: presence, antioxidant and antimicrobial effects. Int J Food Sci Technol.

[CR11] Caliskan O, Radusiene J, Temizel KE, Staunis Z, Cirak C, Kurt D, Odabas MS (2017). The effects of salt and drought stress on phenolic accumulation in greenhouse-grown *Hypericum pruinatum*. Ital J Agron.

[CR12] Chen Z, Ma Y, Yang R, Gu Z, Wang P (2019). Effects of exogenous Ca2+ on phenolic accumulation and physiological changes in germinated wheat (*Triticum aestivum* L.) under UV-B radiation. Food Chem.

[CR13] Donno D, Turrini F (2020). Plant foods and underutilized fruits as source of functional food ingredients: chemical composition, quality traits, and biological properties. Foods.

[CR14] Germ M, Breznik B, Dolinar N, Kreft I, Gaberščik A (2013). The combined effect of water limitation and UV-B radiation on common and tartary buckwheat. Cereal Res Commun.

[CR15] Germ M, Árvay J, Vollmannová A, Tóth T, Golob A, Luthar Z, Kreft I (2019). The temperature threshold for the transformation of rutin to quercetin in Tartary buckwheat dough. Food Chem.

[CR16] Gharibi S, Tabatabaei BES, Saeidi G, Talebi M, Matkowski A (2019). The effect of drought stress on polyphenolic compounds and expression of flavonoid biosynthesis related genes in *Achillea*
*pachycephala* Rech. f. Phytochemistry.

[CR17] Guo R, Shi LX, Jiao Y, Li MX, Zhong XL, Gu FX, Liu Q, Xia X, Li HR (2018). Metabolic responses to drought stress in the tissues of drought-tolerant and drought-sensitive wheat genotype seedlings. AoB Plants.

[CR18] Hajam YA, Lone R, Kumar R, Lone R, Khan S, Mohammed Al-Sadi A (2023). Role of plant phenolics against reactive oxygen species (ROS) induced oxidative stress and biochemical alterations. Plant phenolics in abiotic stress management.

[CR19] Hendrickson PH, Kaufman DA, Lunte EC (1994). Electrochemistry of catehol-containing flavonoids. J Pharm Biomed Anal.

[CR20] Hodaei M, Rahimmalek M, Arzani A, Talebi M (2018). The effect of water stress on phytochemical accumulation, bioactive compounds and expression of key genes involved in flavonoid biosynthesis in *Chrysanthemum*
*morifolium* L. Ind Crops Prod.

[CR21] Hura T, Hura K, Grzesiak S (2009). Possible contribution of cell-wall-bound ferulic acid in drought resistance and recovery in triticale seedlings. J Plant Physiol.

[CR22] Karakaya S, Koca M, Sytar O, Duman H (2021). Determination of natural phenolic compounds of Ferula longipedunculata Peşmen and assessment their antioxidant and anticholinesterase potentials. Nat Prod Res.

[CR24] Kianersi F, Abdollahi MR, Mirzaie-Asl A, Dastan D, Rasheed F (2020). Identification and tissue-specific expression of rutin biosynthetic pathway genes in *Capparis*
*spinosa* elicited with salicylic acid and methyl jasmonate. Sci Rep.

[CR26] Kim NS, Kwon S-J, Cuong DM, Jeon J, Park JS, Park SU (2019). Accumulation of phenylpropanoids in tartary buckwheat (*Fagopyrum tataricum*) under salt stress. Agronomy.

[CR27] Liang N, Kitts DD (2015). Role of chlorogenic acids in controlling oxidative and inflammatory stress conditions. Nutrients.

[CR28] Liu Z, Ren Z, Zhang J, Chuang CC, Kandaswamy E, Zhou T, Zuo L (2018). Role of ROS and nutritional antioxidants in human diseases. Front Physiol.

[CR29] Lucci N, Mazzafera P (2009). Distribution of rutin in fava d'anta (*Dimorphandra*
*mollis*) seedlings under stress. J Plant Interact.

[CR30] Lukšic L, Árvay J, Vollmannová A, Tóth T, Škrabanja V, Trcek J, Germ M, Kreft I (2016). Hydrothermal treatment of Tartary buckwheat grains hinders the transformation of rutin to quercetin. J Cereal Sci.

[CR31] Matsui K, Walker AR (2020). Biosynthesis and regulation of flavonoids in buckwheat. Breed Sci.

[CR32] Mibei EK, Ambuko J, Giovannoni JJ, Onyango AN, Owino WO (2016). Carotenoid profiling of the leaves of selected African eggplant accessions subjected to drought stress. Food Sci Nutr.

[CR33] Mikulajová A, Šedivá D, Hybenová E, Mošovská S (2016). Buckwheat cultivars—phenolic compounds profiles and antioxidant properties. Acta Chimica Slovaca.

[CR34] Moreira X, Abdala-Roberts L, Hidalgo-Galvez MD, Vázquez-González C, Pérez-Ramos IM (2020). Micro-climatic effects on plant phenolics at the community level in a Mediterranean savanna. Sci Rep.

[CR35] Nakabayashi R, Mori T, Saito K (2014). Alternation of flavonoid accumulation under drought stress in Arabidopsis thaliana. Plant Signal Behav.

[CR36] Nisar N, Li L, Lu S, Khin NCh, Pogson BJ (2015). Carotenoid metabolism in plants. Mol Plant.

[CR37] Nouraei S, Rahimmalek M, Saeidi G (2018). Variation in polyphenolic composition, antioxidants and physiological characteristics of globe artichoke (*Cynara*
*cardunculus* var. *scolymus* Hayek L.) as affected by drought stress. Sci Hortic.

[CR38] Raja V, Qadir SU, Alyemeni MN, Ahmad P (2020). Impact of drought and heat stress individually and in combination on physio-biochemical parameters, antioxidant responses, and gene expression in *Solanum*
*lycopersicum*. 3 Biotech.

[CR39] Rice-Evans CA, Miller NJ, Paganga G (1996). Structure-antioxidant activity relationships of flavonoids and phenolic acids. Free Radic Biol Med.

[CR40] Rosero A, Granda L, Berdugo-Cely JA, Šamajová O, Šamaj J, Cerkal R (2020). A dual strategy of breeding for drought tolerance and introducing drought-tolerant, underutilized crops into production systems to enhance their resilience to water deficiency. Plants (basel, Switzerland).

[CR41] Rustioni L, Bianchi D (2021). Drought increases chlorophyll content in stems of *Vitis interspecific* hybrids. Theor Exp Plant Physiol.

[CR42] Šamec D, Karalija E, Šola I, Vujˇci´c Bok, V., Salopek-Sondi, B., (2021). The role of polyphenols in abiotic stress response: the influence of molecular structure. Plants.

[CR43] Sarker U, Oba S (2018). Drought stress enhances nutritional and bioactive compounds, phenolic acids and antioxidant capacity of *Amaranthus* leafy vegetable. BMC Plant Biol.

[CR46] Seca AML, Pinto DCGA (2019). Biological potential and medical use of secondary metabolites. Medicines (basel).

[CR47] Sharma A, Thakur S, Kumar V, Kanwar MK, Kesavan AK, Thukral AK, Bhardwaj R, Alam P, Ahmad P (2016). Pre-sowing seed treatment with 24-epibrassinolide ameliorates pesticide stress in *Brassica*
*juncea* l. through the modulation of stress markers. Front Plant Sci.

[CR48] Sharma A, Yuan H, Kumar V, Ramakrishnan M, Kohli SK, Kaur R, Thukral AK, Bhardwaj R, Zheng B (2019). Cas-tasterone attenuates insecticide induced phytotoxicity in mustard. Ecotoxicol Environ Saf.

[CR49] Sofi SA, Ahmed N, Farooq A, Rafiq S, Zargar SM, Kamran F, Dar TA, Mir SA, Dar BN, Mousavi Khaneghah A (2022). Nutritional and bioactive characteristics of buckwheat, and its potential for developing gluten-free products: an updated overview. Food Sci Nutr.

[CR50] Soviguidi DRJ, Pan R, Liu Yi, Rao L, Zhang W, Yang X (2022). Chlorogenic acid metabolism: the evolution and roles in plant response to abiotic stress. Phyton.

[CR51] Suzuki T, Mukasa Y, Morishita T, Kim S, Woo S, Noda T, Takigawa S, Yamauchi H (2015). Possible roles of rutin in buckwheat plant. Jpn Agric Res Q JARQ.

[CR52] Sytar O, Kosyan A, Taran N, Smetanska I (2014). Anthocyanin's as marker for selection of buckwheat plants with high rutin content. Gesunde Pflanzen.

[CR53] Sytar O, Brestic M, Zivcak M, Tran LS (2016). The contribution of buckwheat genetic resources to health and dietary diversity. Curr Genomics.

[CR54] Sytar O, Švedienė J, Ložienė K, Paškevičius A, Kosyan A, Taran N (2016). Antifungal properties of hypericin, hypericin tetrasulphonic acid and fagopyrin on pathogenic fungi and spoilage yeasts. Pharm Biol.

[CR55] Tajner-Czopek A, Gertchen M, Rytel E, Kita A, Kucharska AZ, Sokół-Łętowska A (2020). Study of antioxidant activity of some medicinal plants having high content of caffeic acid derivatives. Antioxidants (basel, Switzerland).

[CR56] Thurow T (2012) Effect of chlorogenic acid and neochlorogenic acid on human colon cancer cells. Food Science Undergraduate Honors Theses. 2. http://scholarworks.uark.edu/fdscuht/2

[CR57] Vollmannová A, Musilová J, Lidiková J, Árvay J, Šnirc M, Tóth T, Bojňanská T, Čičová I, Kreft I, Germ M (2021). Concentrations of phenolic acids are differently genetically determined in leaves, flowers, and grain of common buckwheat (*Fagopyrum esculentum* Moench). Plants.

[CR58] Vollmannová A, Musilová J, Lidiková J, Árvay J, Šnirc M, Tóth T, Bojňanská T, Čičová I, Kreft I, Germ M (2021). Concentrations of phenolic acids are differently genetically determined in leaves, flowers, and grain of common buckwheat (*Fagopyrum esculentum* Moench). Plants (basel, Switzerland).

[CR59] Wang L, Shan T, Xie B, Ling C, Shao S, Jin P, Zheng Y (2019). Glycine betaine reduces chilling injury in peach fruit by enhancing phenolic and sugar metabolisms. Food Chem.

[CR60] Wang R, Gallant E, Wilson MZ, Wu Y, Li A, Gitai Z, Seyedsayamdost MR (2022). Algal p-coumaric acid induces oxidative stress and siderophore biosynthesis in the bacterial symbiont *Phaeobacter*
*inhibens*. Cell Chem Biol.

[CR61] Xu DP, Li Y, Meng X, Zhou T, Zhou Y, Zheng J, Zhang JJ, Li HB (2017). Natural antioxidants in foods and medicinal plants: extraction, assessment and resources. Int J Mol Sci.

[CR62] Yan K, Cui M, Zhao S, Chen X, Tang X (2016). Salinity stress is beneficial to the accumulation of chlorogenic acids in honeysuckle (*Lonicera*
*japonica* Thunb.). Front Plant Sci.

[CR63] Yang LL, Yang L, Yang X, Zhang T, Lan YM, Zhao Y, Han M, Yang LM (2020). Drought stress induces biosynthesis of flavonoids in leaves and saikosaponins in roots of *Bupleurum chinense* DC. Phytochemistry.

[CR64] Zduńska K, Dana A, Kolodziejczak A, Rotsztejn H (2018). Antioxidant properties of ferulic acid and its possible application. Skin Pharmacol Physiol.

[CR65] Zhang X, Wollenweber B, Jiang D, Liu F, Zhao J (2008). Water deficits and heat shock effects on photosynthesis of a transgenic Arabidopsis thaliana constitutively expressing ABP9, a bZIP transcription factor. J Exp Bot.

